# Understanding factors that promote uptake of HIV self-testing among young people in Nigeria: Framing youth narratives using the PEN-3 cultural model

**DOI:** 10.1371/journal.pone.0268945

**Published:** 2022-06-03

**Authors:** Stacey Mason, Oliver C. Ezechi, Chisom Obiezu-Umeh, Ucheoma Nwaozuru, Rhonda BeLue, Collins Airhihenbuwa, Titilola Gbaja-Biamila, David Oladele, Adesola Z. Musa, Karan Modi, Jessica Parker, Florida Uzoaru, Alexis Engelhart, Joseph Tucker, Juliet Iwelunmor

**Affiliations:** 1 Department of Behavioral Science and Health Education, College for Public Health and Social Justice, Saint Louis University, Saint Louis, Missouri, United States of America; 2 Clinical Sciences Division, Nigerian Institute of Medical Research, Medical Compound, Lagos, Nigeria; 3 Department of Health Management and Policy, College for Public Health and Social Justice, Saint Louis University, Saint Louis, Missouri, United States of America; 4 Global Research Against Noncommunicable Diseases (GRAND), Georgia State University, School of Public Health, Atlanta, Georgia, United States of America; 5 College for Public Health and Social Justice, Saint Louis University, Saint Louis, Missouri, United States of America; 6 Department of Medicine, University of North Carolina at Chapel Hill, Chapel Hill, North Carolina, United States of America; Hebei Provincial Center for Disease Control and Prevention, CHINA

## Abstract

It is important to understand how to frame the formats for promoting HIV self-testing to increase uptake among young people. In this study, we used a culture-centered model to understand the narratives of HIV self-testing preferences among young people in Nigeria. We conducted a crowdsourcing contest to solicit ideas surrounding HIV self-testing promotion among young people (10–24 years) in Nigeria from October to November 2018 as part of the 2018 World AIDS Day event. We received 903 submissions and employed thematic content analysis to evaluate 769 eligible youth narratives. Thematic content analysis of the statements from the youth narratives was guided by the PEN-3 cultural model to examine the positive, existential, and negative perceptions (beliefs and values), enablers (resources), and nurturers (roles of friends and family) of HIV self-testing promotion among young people in Nigeria. Several themes emerged as factors that influence the uptake of HIV self-testing among young people in Nigeria. Specifically, seven themes emerged as perceptions: HIV testing accessibility, stigma reduction, and autonomy (positive); HIV self-testing kit packaging and advertisements (existential); lack of knowledge and increased stigma (negative). Seven themes emerged as enablers: social media, school, and government promotion (positive); gamification and animation (existential); high cost and access to linkage to care services (negative); And seven themes emerged as nurturers: peer, families, and faith-based communities (positive); parents and family-centered approach (existential); and partners and family (negative). Our data suggests that increased awareness around HIV self-testing on current youth-friendly platforms, de-stigmatization of HIV and HIV self-testing, decreased prices for HIV self-testing kits, reliability of testing kits, increased linkage to care services, and promotion of self-testing among family members and the community will be beneficial for HIV self-testing scale-up measures among young people in Nigeria.

## Introduction

### HIV and HIV self-testing among young Nigerians

In 2020, 400,000 young people aged 10 to 24 were newly infected with the human immunodeficiency virus (HIV) [1]. As of 2020, in western and central Africa, there were 200,000 new HIV infections [2, 3]. In Nigeria, demographic determinants such as age, sex, marital status, and education level are associated with HIV testing uptake among adolescents and young adults [4]. Low HIV testing, knowledge of HIV status, sub-optimal treatment, and prevention coverage are vital gaps in the HIV response in sub-Saharan Africa (SSA) among men and young people aged 15 to 24 [5]. This gap compromises the UNAIDS 90-90-90 targets, particularly the first 90, that 90% of all people living with HIV are aware of their status [6]. Given the increasing HIV infection rate among youth aged 15–24 in western and central Africa, challenges remain when accessing HIV and sexual and reproductive health and services among young people globally [7]. These challenges are associated with factors such as sex, gender identity, sexual orientation, behavior, place of residence, and socioeconomic status [7]. The National Agency for the Control of AIDS (NACA) 2016–2020 National HIV Strategy for Adolescents and Young People indicated inadequate HIV knowledge among adolescents and young people in Nigeria [8]. Nationally representative population-based survey data suggests that comprehensive HIV knowledge remain below 50 percent in countries with available data, and gender differences in HIV knowledge may be based on the country’s context [1].

To prevent HIV infections among young people globally, the World Health Organization (WHO) recommends HIV self-testing (HIVST) as an additional approach to HIV testing services [9]. HIVST is a process in which a person collects their own specimen (oral, fluid, or blood) and then performs an HIV test and interprets the result, many times in a private setting or with someone they trust [9]. Evidence suggests that HIVST can be an empowering, discreet, and acceptable option to reach those, including young people, who may not otherwise test [10–12]. Studies also suggest that privacy when testing and regulation over the testing process can motivate young people to self-test for HIV [5, 13]. On the other hand, the potential for social harms, concerns about the validity of HIVST, lack of emotional support when testing alone, and the cost of HIVST kits are cited as barriers to HIVST uptake [14–16].

### HIV self-testing from youth narratives

While there is an emerging body of knowledge on barriers and motivators to HIVST among young people, little effort has been made to understand young people’s perspectives on solutions to address these barriers and improve HIVST rates. Traditional methods of data collection such as surveys, focus groups, and interviews lead the process of engaging young people in discussions about their health [17]. Yet, these methods, and the questions posed, typically are adult-centered and do not explain ways in which young people make sense of their thoughts and opinions [17]. Evidence suggests that youth-centered activities, such as visual storytelling and crowdsourcing, can help promote reflection and communication about issues that can be difficult for young people to express [17–20]. Evidence also suggests that social representations in fictional narratives by young people in African countries provides access to their voices when conceptualizing HIV topics [21, 22]. Further, crowdsourcing involves soliciting solutions to a problem from a large group of people, and then sharing the solutions with the public [23]. Crowdsourcing is low-cost, instant, and can accompany data collection methods like in-depth interviews and focus groups, designed for increased benefits in global settings [24, 25]. Engaging young people in creative activities are instrumental when presenting the complexities of experiences to encourage their openness and expression [17, 26].

### Study purpose

The present study sought to combine the creative narratives of young people within a crowdsourcing contest to promote HIVST uptake in Nigeria. Crowdsourcing is ideal for this study as it allows young people the space and agencies to voice their ideas around HIV prevention challenges while also encouraging responses that better contextualize HIVST among young people in Nigeria. In the context of generative ideas, youth narratives are a source of insight into how young people make sense of HIVST and share this understanding with others. We situate our analyses within the PEN-3 cultural model, which centralizes culture in everyday lay meanings of health [27]. We pay close attention to individual’s perceptions when given supportive spaces to make sense of their health, the structures or opportunities available to enhance health, and the role of key individuals, including family and health systems, in supporting or compromising health behaviors. In this study, the PEN-3 cultural model allows us to explore young people’s creative and spontaneous solutions to promoting HIVST uptake in response to the question: How might we promote HIVST among young people aged 10–24 in Nigeria?

### The PEN-3 cultural model

**Fig 1:**
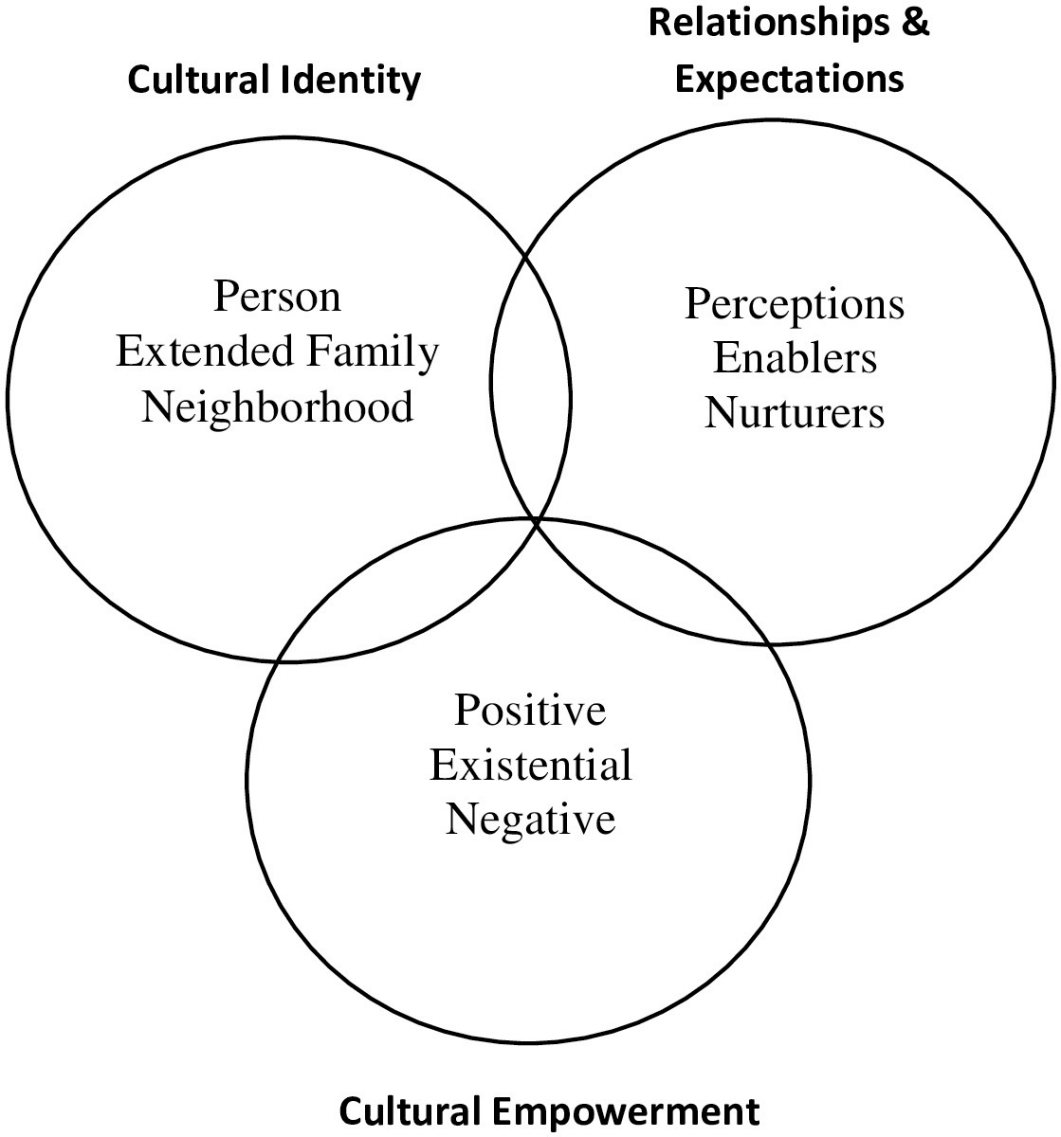
The PEN-3 cultural model [adapted from Iwelunmor et al. [31]].

The PEN-3 cultural model examines health behavior using a collective approach. The model has been used to assess qualitative data through focus group discussions in studies related to health literacy [28] and adolescent self-care performance for Type 1 Diabetes [29]. The model has also been used to understand HIV/AIDS stigma in the South African family and health care settings [27] and explore the influence of culture on African American mothers’ and daughters’ acceptance of the HPV vaccine [30]. The three interrelated domains of the PEN-3 cultural model are as follows: 1) Cultural Identity; 2) Relationships and Expectations; and 3) Cultural Empowerment [31]. Within each domain are three factors that make up the acronym PEN [31, 32] (Fig 1). The Cultural Identity domain includes the factors Person, Extended Family, and Neighborhood, and posits that health interventions should extend beyond the individual and incorporate family and neighborhood contexts as intervention access points and targets to health behavior change [31, 33]. Relationships and Expectations, the second domain, includes Perceptions, Enablers, and Nurturers, and proposes that the formulation and analysis of health problems can be based on the interplay of things (e.g., perception, resources, family, and culture) [31]. Thus, Cultural Empowerment, the third domain, suggests those attributes that are Positive, Existential (unique), and Negative for assessing health problems [31, 34]. For purposes of this study, we focus on the PEN-3 cultural model constructs in the Relationships and Expectations domain and those in the Cultural Empowerment domain to determine HIVST beliefs and values held by people who play instrumental roles in the lives of young Nigerians and the enabling and nurturing factors that influence the promotion of HIVST among young people in Nigeria.

## Methods

We conducted a crowdsourcing contest as part of the Innovative Tools to Expand Youth-Friendly HIV Self-Testing (I-TEST) study, known locally as 4 Youth by Youth (4YBY), in Lagos, Nigeria [35]. From October to November 2018, we solicited ideas from youth concepts and strategies for promoting HIVST.

### Data collection

A crowdsourcing contest was coordinated and implemented as part of the youth-centered and youth-led study team “4 Youth by Youth” (4YBY). The 4YBY team includes young people, health professionals, activists, and entrepreneurs from diverse backgrounds that share a passion for advancing Nigerian youth engagement and leadership in creating innovative and sustainable HIV services. The contest was entitled “The 4 Youth by Youth World AIDS Day HIV Self-Testing Contest,” event in Lagos, Nigeria, which marked the annual global World AIDS Day, celebrated on December first each year. The goal of the contest was to solicit ideas and/or concepts in response to the following key question: How might we promote HIVST among young people in Nigeria? The hashtag “HIVSELFTESTINGCONTEST” was generated to identify the campaign on social media platforms. The research team organized a multi-sectoral contest advisory panel to implement the contest, which consisted of youth representatives from diverse backgrounds and cultures to participate and evaluate ideas based on pre-specified criteria. The contest advisory panel also selected the top 30 finalists and invited them to deliver a three-minute pitch of their idea to a panel of judges at the World AIDS Day event and announced finalists and winners at the end of the event. Participants were recruited through open calls disseminated through social media (e.g., Facebook and Instagram) and in-person announcements and flyer promotions at secondary/high schools, universities, and community centers where young people congregate in Lagos state. In organizing the contest, we used the TDR Practical Guide on Crowdsourcing in Health and Health Research [23]. The guidelines for ethical consent for young persons’ participation in research and access to sexual and reproductive health services in Nigeria were followed for participants’ research consent [36]. Per the guidelines, adolescents aged 13–17 can have access to HIV and sexual and reproductive health (SRH) services without parental consent around research concerning HIV/SRH messaging and education [36]. A detailed account of contest components is included in an earlier qualitative evaluation of the 4YBY HIVST crowdsourcing contest [19]. This research was approved by the Saint Louis University Institutional Review Board in the United States (U.S.) and the Nigerian Institute of Medical Research Ethics Review Board in Lagos, Nigeria.

### Data analysis

De-identified participant entries were transcribed, and thematic data analysis was performed [37, 38]. The entries included written descriptions, images, and videos in response to the crowdsourcing contest key question, “How might we promote HIVST among young people in Nigeria?” Research staff trained in qualitative data methods coded the transcripts and entered data into Microsoft Excel 2016. Initially, the entries were coded by four trained research staff members. Content analysis was conducted to code the data and identify similarities in patterns or categories. An amendable coding sheet, informed by the data, was used to code the entries to identify similarities and differences between the data. After initial coding, the coding sheet was refined by rereading the entries. A sample of the transcripts were provided by two researchers external to the study team who independently reviewed and corroborated themes and coding. When themes and sub-themes were finalized, two research team members conducted a final round of coding the entries. A third researcher confirmed the analysis and resolved any discrepancies with the coding. Descriptive statistics which described participant demographics and submission characteristics were generated through SAS version 9.4. The Consolidated Criteria for Reporting Qualitative Research (COREQ) [39] guided the reporting of the results.

## Results

### Key characteristics

We received a total of 903 entries over a seven-week period, including 353 entries submitted in-person and 550 online submissions. The research team screened and shortlisted entries based on pre-specified eligibility criteria. Entries were excluded due to incompleteness, plagiarism, and not meeting the age criteria (e.g., must be between the ages of 10–24 years at the time of entry). In total, we found 769 eligible, non-duplicated entries submitted by 769 entrants. Table 1. provides an overview of the characteristics of the 769 eligible entrants. There were more entrants (n = 183) between 15 and 19 years, and more than half (51.2%) were females and had obtained a primary education (52.6%).

**Table 1:** Key characteristics of contest entries, HIVST* crowdsourcing contest participants—Nigeria, 2018 (n = 769 eligible entries).

	N	%
**Total**	769	100.0
**Age (years)**		
10–14	139	33.6
15–19	183	44.2
20–24	92	22.2
**Gender**		
Female	376	51.2
Male	358	48.8
**Educational attainment**		
Primary	271	52.6
Secondary	220	42.7
Tertiary	24	4.7

*HIVST = HIV Self-Testing

Note: Some frequencies do not add up to the total due to missing observations.

### The PEN-3 cultural model domains: Relationships and expectations/ cultural empowerment

Consistent with the application of the PEN-3 cultural model, we generated nine cells by crossing the three domains of Relationships and Expectations with the three domains of Cultural Empowerment (see Table 2). A total of 17 themes guided the narratives for the following constructs: positive perceptions of HIVST, existential perceptions of HIVST, negative perceptions of HIVST; positive enablers of HIVST, existential enablers of HIVST, negative enablers of HIVST; and positive nurturers of HIVST, existential nurturers of HIVST, and negative nurturers of HIVST.

**Table 2:** Application of the PEN-3* cultural model.

PEN-3	Positive	Existential	Negative
**Perceptions**	Highly convenient and discreet nature of HIVST *“The HIVST test kit has the potential to increase access to HIV testing*. *It gives one convenience and privacy and it’s very easy to use*. *It can also be beneficial to people who don’t have easy access to healthcare centers…” (#I115)*	HIVST packaging formats, HIV awareness, and HIVST advertisements *“A good kits would be appealing to the eyes and will of the youths*, *it should be designed in beautiful colors*, *cartoon pictures with the first aid logo on it*.*” (#150)*	Lack of knowledge or awareness around HIV and HIVST *“Nigeria has lacked behind in the adoption and creation of awareness of HIV self-testing as a testing option to increase access to HIV testing because of constraints and arguments against HIV self-test*.*” (#057)*
	Reducing HIV-related stigma and discrimination *“The issue of testing and stigmatization is yet a major challenge*, *especially among youth in Nigeria*. *Young people are often not encouraged to seek information on HIV especially from an adult who may be judgmental and discriminatory*. *Worse still people often misjudge others when they want to go for HIV test when it is not for a job requirement or religious body prerequisite for marriage*. *Hence*, *HIV self-testing is a paradigm to overcoming some the drawbacks to gain positive attitudes toward HIV test*.*” (#o56)*		Privacy of results and stigmatization by others when testing for HIV *“say no to HIV stigmatization*, *after the test has been done*, *individuals with HIV should not be treated as an outcast*, *they should not be stigmatized because of this stigmatization most of our youth are afraid to know and check their HIV status*.*” (#321)*
	Autonomy and self-empowerment *“more active role in managing their [youth] health and sexuality” (#I344)*		
**Enablers**	Mobile health strategies *“We can harness the way social media has linked young people all over the world in discussions about issues that directly affect them*. *Spurring interest and awareness of HIV self-testing can lead to conversations about it among young people*.*” (#o367)*	Digital technology, using languages relatable to Nigerian youth, and advertisements *“A key aspect of increasing awareness [of HIVST] would be using cartoon characters to create awareness on HIV and HIV self-testing in English and Pidgin English…” (#248)*	Cost associated with purchasing HIV self-tests *“A lot of people don’t have the money or privilege to for HIV self-testing*.*” (#003)*.
	Locations such as schools, local government townhalls, churches, mosques, youth-friendly centers, concerts, and sports betting centers *“HIV self-testing can be promoted and supported by the government*, *non-governmental organizations*, *religious bodies*, *people should also be sensitized from grass root level e*.*g*., *rural areas*, *students*, *illiterates*, *and others about the importance of knowing their status*.*” (#301)*	Training on the product and partnering with trusted companies *“Companies producing these drugs can partner with authenticated companies producing such toolkits so that controlled access to the equipment is made possible to prevent inefficient or fake toolkits from reaching the hands of youths to avoid turnout of wrong hiv test result*.*” (#169)*	Lack of access to linkage to care services for those who have reactive test results *“Crucial that provision of pre- and post-test counseling should be made available in order to avoid misinterpreting the instructions…people might commit suicide if they received positive HIV self-testing results without formal support from a health care provider or counselor… no referral mechanism to link them to a health facility after self-testing*.*” (#057)*
**Nurturers**	Engaging peers as active change agents *“…Students are the ones that will still go back to their local area*, *home*, *friends*, *mosque or church and people cherish them as student of knowledge a lot and they influence others a lot…” (#48)*	A family-centered approach, in which family members and society work collectively to foster interactions around HIVST *“Teamwork which would involve parents and the society would help in promoting self-testing among youths*, *encouraging the intervention of the kit*.*” (#096)*.	Influence of partners and family *“HIV destroyed the useful facilities in anyone who is contacted and cannot do anything meaningful… tarnishes the image of the family*.*” (#135)*
	Educating family members on HIVST to prevent spread to children and utilizing community gatekeepers *“HIV self testing will also decrease the rate of HIV in families so that mothers who are a victim of this deadly disease virus can know how to breast feed their baby without them contracting the virus and self-testing will promote long life*.*” (#312)*		
	Leveraging the influence of faith-based leaders and faith communities in facilitating the uptake of HIVST *“The citizens revere their religious and believe whatever they hear from their religious leaders as being in their best interests*. *Consequently*, *the religious gatherings can be used as a means of disseminating information’s on HIV self testing*.*” (#58)*.		

*Application of the PEN-3 cultural model by crossing the three domains of relationships and expectations with the three domains of cultural empowerment.

#### Perceptions: Beliefs and values about HIV and HIVST by young people in Nigeria

*Perceptions-positive*. There were a wide range of positive perceptions of HIVST among young people. The highly convenient and discreet nature of HIVST, as well as reduced potential of experiencing stress due to long travel times, meant that providing HIVST would potentially increase access to HIV testing services among individuals who may have limited access to health facilities, as depicted in the following statement:

*“The HIVST test kit has the potential to increase access to HIV testing*. *It gives one convenience and privacy and it’s very easy to use*. *It can also be beneficial to people who don’t have easy access to healthcare centers…”*

*(#I115)*

The possibility of HIVST in reducing HIV-related stigma and discrimination or *“fear of being perceived as a promiscuous person”* (#o28), that are often experienced by young people when accessing HIV testing and/or pre-test information at the health facilities, were frequently cited by the participants, as described below:

*“The issue of testing and stigmatization is yet a major challenge especially among youth in Nigeria*. *Young people are often not encouraged to seek information on HIV*, *especially from an adult who may be judgmental and discriminatory*. *Worse still*, *people often misjudge others when they want to go for HIV test when it is not for a job requirement or religious body prerequisite for marriage*. *Hence*, *HIV self-testing is a paradigm to overcoming some the drawbacks to gain positive attitudes toward HIV test*.*”*

*(#o56)*

The added autonomy and self-empowerment conferred by HIVST was perceived to be beneficial in creating a *“more active role in managing their [youth] health and sexuality” (#I344)*. A few participants believed that HIVST also creates an opportunity to reach Nigerian youth who may not have otherwise tested for HIV and increase HIV retesting uptake among individuals at higher risk of HIV acquisition.

*Perceptions-existential*. The existential perceptions of HIVST among young people centered around HIVST packaging formats, HIV awareness, including HIVST advertisements. Testing kit package instructions, package appeal, and package names were often cited to promote adoption and discretion when testing. In relation to this, a participant mentioned the following:

*“Test kit can be packaged in a cotton*, *form*, *the size be in form of a thermometer like form which comes with a pack or sterilized needle…small innovating book to give them the courage that there is still hope in life*.*”*

*(#193)*

Advertisements were mentioned when promoting HIVST and increasing the public’s perception. This can be seen in the following statement:

*“One of such ways is by placing detailed advertisements on radio programs*, *television stations*, *and newspapers; explaining vividly on the importance and benefits of this self-test*, *as well as how to carry it out*.*”*

*(#294)*

*Perceptions-negative*. The negative perceptions related to HIVST were related to ignorance surrounding HIV and fear when testing. Lack of knowledge or awareness around HIV and HIVST was expressed in the following statement:

*“Nigeria has lacked behind in the adoption and creation of awareness of HIV self-testing as a testing option to increase access to HIV testing because of constraints and arguments against HIV self-test*.*”*

*(#057)*

Contrary to positive perceptions around reducing HIV-related stigma and discrimination when promoting HIVST, some negative perceptions around HIV and HIVST also touched on stigmatization and were often centered around privacy of results and typecasting an individual with HIV as an “outcast” (#*321*). Stigmatization by others when testing for HIV and fear of one’s status being compromised were frequently noted:

*“say no to HIV stigmatization*, *after the test has been done individuals with HIV should not be treated as an outcast*, *they should not be stigmatized because of this stigmatization most of our youth are afraid to know and check their HIV status*.*”*

*(#321)*

#### Enablers: Resources that either promote or hinder efforts when promoting HIVST among young people in Nigeria

*Enablers-positive*. Participants identified various mobile health strategies as enablers when promoting HIVST, reaching other young people, and connecting young people to appropriate sexual health services and support systems. Other participants cited governmental, community, and school resources as positive enablers. Due to the extensive use of smartphones and widespread internet access among young people, majority of participants cited the use of social media platforms and mobile apps, like WhatsApp, Facebook, Instagram, and Twitter to increase awareness of HIVST and maintain visibility online, as illustrated below:

*“We can harness the way social media has linked young people all over the world in discussions about issues that directly affect them*. *Spurring interest and awareness of HIV self-testing*, *can lead to conversations about it among young people*.*”*

*(#o367)*

Participants also identified certain locations that would be beneficial in making HIVST more accessible to young people in the community. The most frequently cited location for promoting HIVST was in the school setting. One participant suggested introducing HIV prevention in school curriculums and including information on HIVST:

*“Moreover*, *adding HIV self-testing into school syllabus will be a great thing because both students in primary and secondary school will be a beneficiary of it*. *This HIV self-testing should be added to civic education in school because it is a core subjects*. *This will help the students to know their HIV status and be able to disseminate It to their parent at home*, *also establishing social clubs known as society or curriculum activity in school*, *Those social club will serve as counseling link between the doctor and the students through the social club master who will deliver the practical aspect of HIV self-testing after the students had been taught the theory aspect in their various classes*. *Those club masters will also be a public awarer that will go to various places like markets*, *universities and colleges of education to teach people the practical aspect of HIV self-testing*.*”*

*(#184)*

Other convenient and accessible community venues were also mentioned, such as local government town halls, churches, mosques, youth-friendly centers, concerts, and sports betting centers.

*Enablers-existential*. Like the earlier themes, existential enablers by young people were related to the use of digital technology and advertisements when promoting HIVST. A less common but related theme was incorporating elements of games (gamification) to enhance audience engagement while providing health information and critical measures related to HIVST:

“*It is a story/adventure game in which players would be able to control the actions and choices of characters revolving around real HIV/AIDS issues among youth*, *suggesting regular self-testing*. *VIDA will come with an online community where youth from all over the country can discuss issues and questions with the input of professionals*.*”*

*(#62)*

Participants also noted the need to be trained on the product and partnering with trusted companies when promoting the HIVST toolkit, as described below:

*“Companies producing these drugs can partner with authenticated companies producing such toolkits so that controlled access to the equipment is made possible to prevent inefficient or fake toolkits from reaching the hands of youths to avoid turnout of wrong hiv test result*.*”*

*(#169)*

*Enablers-negative*. There were concerns about decentralizing HIV testing by promoting self-testing beyond facility-based sites. Negative enablers were related to the cost associated with purchasing HIV self-tests and the lack of access to linkage to care services for those who have reactive test results. The first, cost associated with HIVST and facilitating uptake, was described in the below statement:

*“The cost of an HIV self-test kit is a potential barrier to adoption*, *willingness to use*, *purchase*, *and the uptake of the HIV self-test*, *particularly among people in poor resource settings… not willing to purchase the self-testing kits as a result of the cost*.*”*

*(#057)*

Thus, most participants expressed concerns with those who undergo HIVST having little to no access to pre or post-test counseling support, including linkage to care and treatment services that are typically provided by in-person test counselors:

*“Crucial that provision of pre- and post-test counseling should be made available in order to avoid misinterpreting the instructions…people might commit suicide if they received positive HIV self-testing results without formal support from a health care provider or counselor… no referral mechanism to link them to a healthy facility after self-testing*.*”*

*(#057)*

#### Nurturers: Role of friends and family in promoting HIVST among young people in Nigeria

*Nurturers-positive*. Participants emphasized the importance of engaging their peers as active change agents in promoting the uptake of HIVST in their communities. They also mentioned educating family members on HIVST to prevent spread to children and utilizing community gatekeepers. A participant mentioned:

*“…Students are the ones that will still go back to their local area*, *home*, *friends*, *mosque or church and people cherish them as student of knowledge a lot and they influence others a lot…”*

*(#48)*

A few participants suggested leveraging the influence of faith-based leaders and faith communities in facilitating the uptake of HIVST. A participant stated:

*“The citizens revere their religious and believe whatever they hear from their religious leaders as being in their best interests*. *Consequently*, *the religious gatherings can be used as a means of disseminating information’s on HIV self testing*.*”*

*(#58)*

*Nurturers-existential*. The existential nurturers included parents and the family at large in HIV discussions. One participant noted the lack of such discussion due to traditions and norms:

*“Our tradition*, *norms*, *and values do not promote discussion on sexuality between parents and their children*.*”*

*(#338)*

Further, a participant suggested a family-centered approach, in which family members and society work collectively to foster interactions around HIVST, as illustrated:

*“Teamwork which would involve parents and the society would help in promoting self-testing among youths*, *encouraging the intervention of the kit*.*”*

*(#096)*

*Nurturers-negative*. The influence partners and family have when learning about HIV and promoting HIVST was highlighted in the narratives:

*“Unfavorable reaction of partners in relationships and marriages also make people scared of knowing their status especially when the kit can show their path at their comfort zones*.*”*

*(#058)*

*“HIV destroyed the useful facilities in anyone who is contacted and cannot do anything meaningful… tarnishes the image of the family*.*”*

*(#135)*

Furthermore, participants noted the unfavorable reaction of partners when testing for HIV and the impact HIV has on the family’s image.

## Discussion

For this study, we used the PEN-3 cultural model to better understand context-specific dynamics around HIVST promotion among Nigerian youth. Findings reveal cultural resources available to young people as they strive to make sense of HIVST and identify context-specific distinctions and nuances necessary for creating demand, improving access, and reaching young Nigerians with HIV who remain undiagnosed. This study enhances the literature by incorporating young people’s voices and narratives to better understand HIV and HIVST uptake in low-and middle-income countries (LMICs) and by broadening the reach of HIVST in this age group.

Positive perceptions around HIVST centered around preferences young Nigerians had for the confidential nature of HIVST and the convenience that HIVST provides in permitting testing at home and eliminating travel times for HIV testing in health facilities. This finding is consistent with other studies that have been conducted globally, and in SSA, that have researched HIVST uptake and acceptability [40, 41]. Study participants mentioned that HIVST allows for decreased stigma and autonomy when testing, which can be beneficial in expanding HIVST reach. Prior research has mentioned that stigma and confidentiality concerns were barriers contributing to uptake of HIV testing services in SSA [42–44]. Our findings suggest potential for increased uptake of HIVST among young people in Nigeria if stigma-related barriers are decreased and youth feel empowered to test in the comfort of their environments. Stigma-related barriers around HIV and HIV testing can be decreased by improving HIV knowledge among young people in Nigeria and educating young Nigerians about HIV and the benefits of HIV testing [4]. Furthermore, self-testing done privately or with a trusted individual can avoid threats in confidentiality [16], which could allow for young people to feel empowered to test more frequently or in environments they trust.

Existential perceptions among young people were related to kit packaging and HIVST advertising. Studies have noted that increased anonymity and incorporation of innovative user preferences with packaging will be required when reducing stigma-related barriers and appealing to those of various literacy or education levels [40, 41]. Youth from our study mentioned how television, radio, and newspaper advertisements could be used to encourage other youth to utilize HIVST. Evidence from one study suggests that Quick Response (QR) codes from print advertisements directed 13% of visitors to the study’s website when assessing the feasibility of a youth-driven social media campaign to promote increased knowledge and testing for sexually transmitted infections (STIs) and HIV among adolescents in Philadelphia [45]. This relates to our findings by incorporating innovative techniques to promote HIVST, like the use of QR codes on print advertisements such as newspapers, or when strategizing about things that attract youth for promotional items and those suited for television or radio formats. Lack of awareness around HIV and persistent stigma of HIV were often cited as negative perceptions.

Our study also identified positive, existential, and negative enablers for HIVST promotion among young people in Nigeria. A positive enabler included using social media platforms, while an existential enabler included using gamification to promote relatable storytelling and create entertaining and informative ideas to promote HIV and HIVST. The implementation of more media-based interventions for youth-engaged HIV testing is consistent with evidence suggesting a youth-driven approach in designing and implementing a campaign using the media and community outreach for HIV/STI testing [45]. The promotion of HIVST by the government and the incorporation of HIVST in school curriculums were also presented as positive enablers. There has been limited literature focusing on governmental support of HIVST, specifically for young people, in promoting awareness, destigmatizing services, and leveraging resources in Nigeria. The United States President’s Emergency Plan for AIDS Relief (PEPFAR) and the Global Fund to Fight AIDS, Tuberculosis, and Malaria provides funding for the HIV response in sub-Saharan Africa, including HIV treatment and testing [46]. This is noteworthy as it can provide additional support for HIVST and utilization in LMICs like Nigeria. Thus, evidence suggests that HIV testing is performed for free in government clinics in Nigeria [47], which may explain the frequent mention of governmental support for HIVST at little to no cost.

As suggested in our results, incorporating HIVST in places most visited by young people has the power to shift the way young people view HIVST and can help with HIVST acceptability. Results from one study looking at the acceptability of HIVST among students in the Democratic Republic of Congo revealed that unsupervised HIVST was 81% more acceptable among those 24 years and younger and associated with prior knowledge of HIVST [48]. A small portion of young people had concerns about the authentication of HIVST toolkits, another existential enabler. Studies have assessed HIVST uptake in key populations with evidence of confirmatory testing [49–51]. For example, one study estimated a 56% timely linkage of confirmatory testing and HIV care following HIVST [51], while another reported a relatively low number of confirmatory tests [49]. Although our study shows some concern about HIVST authenticity, confirmatory tests, and linkage to care activities for young people utilizing HIVST, will benefit long-term use and test kit reliability.

Young Nigerians expressed concerns relating to high costs for the HIVST toolkits. There have been new pricing deals of HIVST and additional suppliers to make HIVST more accessible through a Unitaid-led pricing agreement [52]. Our findings inform studies that have conducted HIVST among young people, and adults, when conveying preferences around HIVST and providing kits at little to no costs [13, 47, 53]. Like concerns related to the authenticity of HIVST, participants also expressed concerns with pre and post-test counseling services for those with reactive HIVST results. This finding suggests the need to provide additional emotional and mental health support services and linkage to care services for youth who test for HIV and those who test positive. Studies have explored emotional, behavioral, and psychosocial contexts for youth living with HIV [54, 55]. Among Tanzanian adolescents living with HIV, self-blame and lack of acceptance around their HIV-positive status presented challenges, while social support and social networks composed of other persons living with HIV/AIDS provided more acceptance with their diagnosis [55]. Additional HIVST and mental health interventions targeting SSA young people who live and do not live with HIV can increase HIV testing promotion and encourage effective strategies for managing emotions upon receiving results. Connecting youth to linkage to care services when conducting HIVST will help facilitate discussions around coping with test results and provide resources for treatment services [16, 56]. More so, reviewing SSA young people age of consent to HIV testing services policies, especially as it relates to age of consent for SSA young people under 16 years of age who want to engage in HIV testing services should be considered when promoting HIVST [57, 58].

Our study also successfully outlined nurturing factors when promoting HIVST. Positive nurturers included involving Nigerian youth as change agents when promoting HIVST to other young people, promoting HIVST among families and shaping mothers’ awareness of prevention measures, and distributing HIVST information in community settings like faith-based institutions. Utilizing youth as change agents when promoting HIVST will be very important when reaching other young people. In one study, 81% of young women in rural South Africa who used HIVST reported talking to their partner after using HIVST and distributed kits to their female friends and family [59]. This informs our findings by showcasing the power of peer-modeling and having young people take an active role in promoting HIVST when engaging with other young people. Further, the promotion of HIVST in faith-based communities from our study is consistent with another study conducted in North Carolina among African Americans. Evidence from the study suggests the influence faith leaders have in their community when modeling health behaviors, like HIV testing [60].

Additionally, parents acted as existential enablers in our study. Among adolescents and young adults in Kenya, evidence suggests that peers, partners, and parents can play important roles in testing decisions [61]. This suggests the need for more family-centered approaches around HIVST to increase acceptability and enhance communication. The influence of partners and family were negative enablers in our study. Participants noted that unfavorable reaction of partners increases fear of knowing one’s status and HIV can tarnish the family’s image. Evidence suggests that a high proportion of pregnant and postpartum women in Kenya, as well as female sex workers, distributed self-tests to their sexual partners and resulted in a high level of couples counseling [62]. This is noteworthy because while promotion of HIVST can be modeled through peers, and distributed through peers, increased research investigating the reach of partners for those using HIVST, and the impact it has on family structures in SSA, will be impactful for increased promotion of HIVST.

While this study has strengths in applying the domains and constructs from the PEN-3 cultural model, it is not without limitations. For one, the PEN-3 cultural model is a rich model in that it uses different domains to examine culture. By focusing solely on the Relationships and Expectations and the Cultural Empowerment domains for the youth narratives, and not the Cultural Identity domain, this study may lack the ability to explore additional context-specific dynamics from youth narratives. Furthermore, because this study was conducted in Nigeria, much of the youth narratives are specific to young people in Nigeria and may lack generalizability with other young people in SSA and their thoughts around HIVST promotion.

## Conclusion

The PEN-3 cultural model is useful when exploring cultural context among Nigerian youth narratives and when promoting HIVST. Additional youth-driven interventions, those aware of culture, and those that consider the unique perspectives of youth, will be helpful when reaching more young people and sustaining HIVST practices. It is important that young people in Nigeria are aware of the agency they possess and their ability to promote HIVST in various settings. This not only shapes the way they view their health but is instrumental in the creation of prevention measures for their communities at large. Family, friends, and community organizations can work together with young people and help with promotion activities. Additional strategies when promoting HIVST among young people in Nigeria should consider accessibility, feasibility, desirability, sustainability, and normalization of HIVST activities.

## Supporting information

S1 File The 4 youth by youth HIV self-testing crowdsourcing contest: A qualitative evaluation. (PDF)

S2 File Strategies for enhancing uptake of HIV self-testing among Nigerian youths: A descriptive analysis of the 4YouthByYouth crowdsourcing contest. (PDF)
